# A Novel Design of a Portable Birdcage via Meander Line Antenna (MLA) to Lower Beta Amyloid (Aβ) in Alzheimer’s Disease

**DOI:** 10.1109/JTEHM.2025.3559693

**Published:** 2025-04-10

**Authors:** Felipe Perez, Jorge Morisaki, Haitham Kanakri, Maher Rizkalla, Ahmed Abdalla

**Affiliations:** IU School of MedicineIndiana University Indianapolis IN 46202 USA; REMFS LLC Indianapolis IN 46278 USA; Electrical and Computer Engineering DepartmentPurdue University 311308 Indianapolis IN 46202 USA

**Keywords:** Alzheimer disease treatment, portable birdcage, S-parameter, SAR, meander line

## Abstract

Late Onset Alzheimer’s Disease (LOAD) is the most common cause of dementia, characterized by the deposition of plaques primarily of neurotoxic amyloid- $\beta $ ( $A\beta $) peptide and tau protein. Our objective is to develop a noninvasive therapy to decrease the toxic A $\beta $ levels, using repeated electromagnetic field stimulation (REMFS) in the brain of Alzheimer’s disease patients. We previously examined the effects of REMFS on $A\beta $ levels in primary human brain (PHB) cultures at different frequencies, powers, and specific absorption rates (SAR). PHB cultures at day in vitro (DIV7) treated with 64 MHz with a SAR of 0.6 W/Kg, one hour daily for 14 days (DIV 21) had significantly reduced (p =0.001) levels of secreted $A\beta $-42 and $A\beta $-40 peptide without evidence of toxicity. The EMF frequency and power, and SAR levels used in our work is utilized in MRI’s, thus suggesting REMFS can be further developed in clinical settings to lower ( $A\beta $) levels and improve the memory in AD patients. These findings and numerous studies in rodent AD models prompted us to design a portable RF device, appropriate for human use, that will deliver a homogeneous RF power deposition with a SAR value of 0.4-0.9 W/kg to all human brain memory areas, lower ( $A\beta $) levels, and potentially improve memory in human AD patients.The research took place at the Indiana University School of Medicine (IUSM) and Purdue University Indianapolis. The first phase was done in PHB cultures at the IUSM. Through this phase, we found that a 64 MHz frequency and an RF power deposition with a SAR of 0.4-0.6 W/kg reduced the (A $\beta $) levels potentially impacting Alzheimer’s disease. The second phase of the project was conducted at Purdue University, we used ANSYS HFSS (High Frequency Simulation System) to design the devices that produced an appropriate penetration depth, polarization, and power deposition with a SAR of 0.4-0.9 W/kg to all memory brain areas of several numerical models. In Phase II-B will validate the device in a physical phantom. Phase III will require the FDA approval and application in clinical trials.The research parameters were translated into a designed product that fits comfortably in human head and fed from an external RF source that generates an RF power deposition with a SAR of 0.4-0.9 W/kg to a realistic numerical brain. The engineering design is flexible by varying the leg capacitors of the Meander Line Antenna (MLA) devices. Thermal outcomes of the results guarantee less than 0.5 C temperature increase within one-hour time of exposure, which can be used in clinical trials for AD patients. Design parameters include dimension of the coil, the MLA structure, conducting material, and capacitance values with the produced EM fields. The flexible design was achieved by varying the additive capacitance between conductors, and via a hybrid approach integrating a birdcage with sixteen MLA. A coil antenna size within 16 cm radius and 13 cm length was achieved. A capacitance between 6.9 nF and 9.2 nF were observed when copper materials with 16 conductors were used to achieve the research parameters in focus.The medical project proposed here has three phases: The initial phase of determining the research parameters for reducing A $\beta $ levels in human brain cultures and animal studies was completed at the IUSM. The translational engineering design of the REMS device and the numerical head and Antenna devices was successfully completed and presented in this paper by Purdue University and IUSM. Future phases will require manufacture and experimental validation of the REMS device with FDA approval for human application. Clinical impact: Our biological studies in human brain cultures showed that an RF power with a SAR of 0.4-0.9 W/kg at 64 MHz, lowered A $\beta $ levels, which potentially will prevent the death of the brain neurons and improve memory in AD. The fact that we found a safe RF power deposition with a SAR value associated with the proposed biological effects in human neurons and that 64 MHz provides a penetration depth of 13.5 cm that reaches all memory areas in a human brain makes the design and manufacture of this device of high clinical impact in the study of these exposures on the treatment of Alzheimer’s and other protein associate diseases. Also, 64 MHz and RF power deposition with similar SAR levels are administer routinely in routine MRI for more than 4 decades makes it a safe framework for these novel therapeutic strategy.Clinical and Translational Impact Statement: The basic science work presented previously is both mechanistic and translational, and would advance the field of neuroscience as well as AD. This prompted us to joint efforts between the Indiana University School of Medicine and the electrical and computer engineering at Purdue University to design and develop a suitable EMF device for human treatments. Recently, our engineering team designed a birdcage antenna that generate a homogeneous RF power deposition with the same SAR values of our biological experiments in a realistic numerical human brain. Here, the engineering research has been extended to investigate the design of a portable flexible birdcage antenna that will enable adjustments to fit physical patient’s characteristics, such as geometry, head size, and tissue dimensions. This new device is expected to improve SAR uniformity and may reduce the likelihood of untreated regions in the brains of patients during treatments. Also, here we determined that the maximum temperature rise of these exposures was less than 0.5°C, which is a safe level per regulatory agencies. This study considers a portable device system that will achieve the research parameters and patient satisfaction regarding reliability and comfort.

## I. Introduction

Alzheimer’s disease (AD) is the most common neurodegenerative dementia worldwide. In 2022, AD cost the nation ${\$}$321 billion, including ${\$}$206 billion in Medicare and Medicaid. Unless we develop an effective treatment, costs will increase by nearly ${\$}$1 trillion by 2050 [1]. AD is characterized by the accumulation of the toxic $\beta $-amyloid $(A\beta)$ in the brain, a key factor in AD pathology [2], [3]. No effective therapy for AD currently exists due to the difficulties of reducing the levels of the toxic beta-amyloid $(A\beta)$ proteins in the brain without triggering brain swelling or microhemorrhages associated with monoclonal antibody therapy for this condition.

The standard of care for AD treatment includes cholinesterase inhibitors, NMDA receptor antagonists, and monoclonal antibodies (mAbs). However, these treatments are ineffective in improving cognition, unable to change disease progression [4], limited in number of therapeutic targets [5], [6], cause severe side effects (brain swelling, microhemorrhages with mAb) [7], lack of understanding of the aging effects on AD [8], and unable to cross the blood-brain-barrier (BBB) effectively [9] to reach all affected brain areas in AD [10]. mAbs are available to lower $(A\beta)$, but their severe side effects make the risk-benefit profile of mAbs unclear [11], [12]. There is an urgent need to develop a safe and effective therapy to cross the BBB, reach the multiple therapeutic targets in the brain, and act on both the aging and AD pathways to lower $(A\beta)$ and stop disease progression. A novel and safe non-invasive multitarget strategy utilizing repeated electromagnetic field stimulation (REMFS) lower $(A\beta)$ levels, stop disease progression [13] and act both on the aging [14] and AD pathway [15], [16] in all memory areas, prevent neuronal death, and improve memory without brain swelling in AD mice [13]. This treatment has not been developed for humans because current EMF devices have poor penetration depth and SAR distribution in the human brain. Thus, it is ineffective in reaching deep memory areas affected early in AD.

Previous REMFS studies at high RF power deposition (900-2000 MHz) with a specific absorption rate (SAR) of 0.25 to 5 Wkg [17], [18] stopped AD progression [19] by lowering $(A\beta)$ levels [2], without causing brain swelling or hemorrhages in numerous AD rodent studies [2], [19], [20], [21], [22], [23], [24], [25], [26], [27], [28], [29]. REMFS not only prevents cognitive impairment but also reverses it [13], [30]. These exposures did not cause any cancer after two years of treatment [13], the main side effect was a body temperature rise (TR) of 1.3° in the AD mice [21], since RF heat can cause tissue injury, it must be kept to a safe level of less than 0.5° per regulatory agencies. Our biological team found that PHB cultures (DIV7) treated at 64 MHz, for one or two hours for 14 days also produced significantly lower $(A\beta)$ levels. Also PHB cultures (DIV28) treated with 64 MHz one hour/day during 4 or 8 days produced a similar significant reduction in $(A\beta)40$ levels. We found that 0.4 W/Kg was the minimum SAR required to produce a biological effect (MSBE), this exposure did not result in cellular toxicity. An RF power deposition with a specific absorption rate (SAR) of 0.4-0.9 WKg) was the minimal effective dose that activates autophagy to degrade and lower $(A\beta)$ in primary human brain cultures. These findings led us to design a prototype device that will homogeneously deliver the same SAR level to all memory areas of a human brain, lower $(A\beta)$ levels, and potentially improve memory in human AD. This study is highly significant because here we designed the first safe and effective portable/wearable birdcage with sixteen meander line antenna legs for Alzheimer’s disease treatments with a frequency that homogeneously reaches deep memory areas with the required SAR level with a temperature rise less than 0.5 degree Celsius per ICNIRP [31], [32]. The next phase will manufacture a device prototype with personalized AI treatments and temperature control and determine its feasibility, safety, and user acceptability for AD. The paper details the design and simulation results leading to the specific SAR, frequency, and linearity required for optimal EMF exposure. It also shows that the 64 MHz frequency has excellent skin depth penetration, allowing it to effectively penetrate multiple head tissue layers, such as hair, skin, fat, dura, cerebrospinal fluid (CSF), and grey matter, reaching deep into the brain areas [33].

**FIGURE 1.**
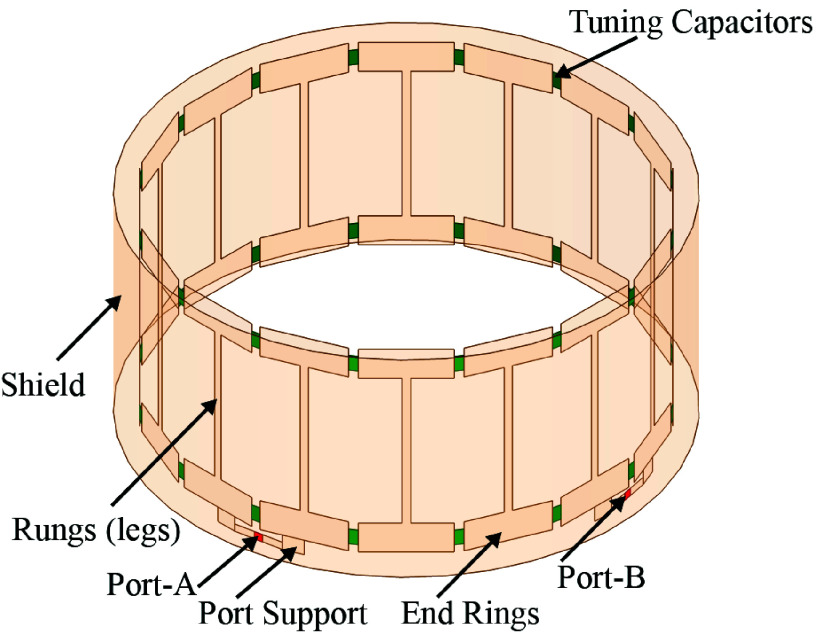
Typical MRI Birdcage coil in high-pass configuration includes 16 rungs and 32 tuning capacitors, with two ports positioned at a 90° displacement from each other.

The birdcage coil stands as a fundamental RF-transmit device in clinical magnetic resonance imaging (MRI) [34]. It’s design, featuring radial and axial conductive elements, enables uniform radio frequency pulse transmission essential for signal reception and excitation during treatments, which achieves near-optimal RF field B1 homogeneity and high signal-to-noise ratio (SNR) [34]. The birdcage coil depicted in Figure 1 comprises two circular conductive loops known as end rings linked by an even number of straight conductive elements referred to as rungs or legs [34]. The number of rungs in birdcage coils is adaptable based on coil size, typically ranging from 8 to 32, where the body coils are typically longer than the head coils [35]. Capacitors strategically placed between conducting elements offer flexibility in achieving desired frequency characteristics, allowing for either a low-pass or high-pass configuration in the birdcage coil design [36]. In clinical MRI, a common setup involves a high-pass configuration with paired capacitors positioned along the end rings [36]. This arrangement emulates a continuous conducting surface, enhancing the coil’s efficiency for radio frequency transmission and reception [34]. The Birdcage device has exhibited considerable promise in AD treatment, as evidenced by numerous previous studies. In a recent paper [33], authors focused on a numerical analysis of an innovative EMF Birdcage portable device designed for the treatment of AD. Specifically, the device was engineered to emit a frequency of 64 MHz and achieve a Specific Absorption Rate (SAR) of $0.6~W/Kg$, directed towards the stimulated human brain.

The article offers a comprehensive study of the proposed device, providing detailed insights into its modeling, encompassing dimensions, port configurations, power levels, and tuning capacitor values. We utilized a sophisticated multi-layer human body model to validate the device’s field distribution in a human head, accounting for specific electrical and thermal properties across brain and body layers. The study conducts a rigorous series of electromagnetic simulations leveraging Ansys HFSS, Q3D Extractor, Circuits and IcePak. These simulations are crucial in evaluating and refining the proposed design, ensuring its efficacy and suitability within the intricate geometry and dynamics of the human anatomy and physiology.

Following this introduction, the article is organized into several key sections to provide a comprehensive overview of the study. Section II describes the electromagnetic field exposures and treatment conditions used for human neuronal cultures. Section III outlines the methods for preparing primary human brain (PHB) cell cultures. Section IV presents the results of neuronal exposures to repetitive electromagnetic field stimulation (REMFS). Section V introduces the proposed birdcage coil model, followed by Section VI, which details the human body and brain models used in the simulation. Section VII discusses the simulation results and key findings. Section VIII provides a comparison between the proposed antenna design and both two-port and eight-port configurations. Section IX focuses on temperature estimation based on the proposed MRI coil, while Section X addresses the limitations of the study and suggests directions for future work. Finally, the conclusion summarizes the overall contributions and implications of the study. An appendix is included to provide additional clarification on the definition and relevance of Specific Absorption Rate (SAR) within the context of this research.

## II. Electromagnetic Field Exposures and Treatment Conditions of Human Neuronal Cultures

We performed the Electromagnetic field exposures using a vertically-mounted IFI TEM Cell (Transversal Electromagnetic Cell, model CC110-SPEC, DC to 1,000 MHz, Test Equipment Corporation, Mountain View, CA, IFI Ronkonkoma NY) [16]. This chamber is an expanded coaxial transmission line operating in the TEM mode, consisting of a main rectangular waveguide that contains a flat-metal-strip center conductor located in the middle between the top and bottom walls. The wall and center conductor are tapered at both ends to provide 50-Ohm impedance along the entire length of the chamber. One port was connected to the RF source (HP 8656B/57A/57B synthesized signal generator) via coaxial cable and the other end to a matched load impedance of 50-ohms (provided by an oscilloscope), which is the characteristic impedance to mimic free space or plane wave irradiation. The complete array was mounted on a compact and portable cart. The wave impedance throughout the chamber is the 377-ohms intrinsic impedance of free space.

## III. Methods for Primary Human Brain (PHB) Cell Cultures

The protocol was approved by the Indiana University School of Medicine Institutional Review Board (IRB) [16]. Primary cultures of mixed human fetal brain (HFB) cells were prepared from the brain parenchyma of aborted fetuses (80-110 days gestational age), as described previously [37]. The meninges and blood vessels were removed; the brain tissue was washed in minimum essential medium and enzymatically dissociated by incubation in 0.05% Trypsin- 0.53 mM EDTA solution at $37^{\circ }C$ in a shaking water bath set to 150 RPM. Tissue was subsequently mechanically dissociated by trituration through a siliconized (Sigma-Cote; Sigma-Aldrich, St Louis, MO), fire-polished Pasteur pipette. Cells were then centrifuged at 800 x g for 10 min, resuspended and seeded at an initial density of $2.2\times 105$ cells/cm2 in Neurobasal (plus GlutaMAX, B27, antibiotic cocktail, normocin, bFGF) and allowed to attach overnight in poly-D-lysine coated 24-well tissue culture plates. The following day, media and non-cellular debris were aspirated from the plate and media replaced with Neurobasal medium (Invitrogen), supplemented with 1x B27, 0.5 mM GlutaMAX, 5 ng/ml basic FGF (Invitrogen), and antibiotic/antimycotic mixture. Half-media changes were performed every 3rd day of culture. Primary human brain cultures have been shown previously to comprise approximately 60 to 70% neurons with 30 to 40% mixed glial cells [38].

## IV. Results of Neuronal Exposures to REMFS

Results in CM samples revealed a 46% reduction of $(A\beta)40$ levels when cultures were subjected to REMFS at 64 MHz with a SAR of 0.6 W/Kg daily for one hour for 14 days and a corresponding 36% reduction in $(A\beta)42$. Additional modifiable variables, such as exposure time and frequency were also considered, and the impact of these different EMF settings was studied relative to the reduction in $(A\beta)40$ and $(A\beta)42$ levels [16]. We also treated PHB cultures differentiated for 28 days to determine if REMFS also reduced $(A\beta)$ levels in cells near the end of primary culture lifespan [38]. Results revealed REMFS at 64 MHz with SAR of 0.9 W/Kg daily for 1 hour after 4 and 8 days produced a significant reduction of $(A\beta)40$ levels in the media cultures. Interestingly, a SAR of 0.4 W/kg produced similar results, although a significant reduction of the $(A\beta)42$ levels was only noted at day 8. Nevertheless, an overall shorter treatment duration also reduced $(A\beta)$ levels (4 or 8 vs. 14 or 21 days). This is an advance from our prior results following 21 days of exposure, leading us to believe that through additional fine tuning of REMFS settings, the desired biological effects of REMFS may ultimately be achieved after only a few treatments. REMFS studies with SAR of 0.25-1.05 W/kg similar to our study with SAR values of 0.4-0.9 EMF frequency and power used in our work were used in AD mouse studies with improvement in memory and Ad brain pathology, thus suggesting REMFS can be further developed in clinical settings to modulate $(A\beta)$ deposition.

## V. Proposed Birdcage Coil Model

RF birdcage coils typically comprise two circular conductive loops known as end rings, N conductive straight elements called rungs (or legs), and capacitors positioned on either the rungs, end rings, or both. The placement of these capacitors within the coil geometry categorizes birdcage coils into three types: low-pass, high-pass, and band-pass configurations, each tailored to specific frequency filtering characteristics [36]. For instance Figure 1 shows a typical MRI Birdcage coil in high-pass configuration includes 16 rungs and 32 tuning capacitors, with two ports positioned at a 90° displacement from each other.

Within an RF birdcage coil featuring N legs and capacitors of uniform values, there emerge N/2 distinct resonant modes [36]. Among these modes, the $m=1$ state signifies either the lowest frequency mode in low-pass birdcage coils or the highest frequency resonant mode in high-pass configurations [36]. This particular $ m=1$ mode holds paramount importance in MRI applications as it generates a highly uniform $B1$ field within the coil, indispensable for achieving precise and reliable imaging outcomes [36]. The inductance matrix given below is derived using Ansys Q3D Extractor, and it represents the self and mutual inductances of the proposed coil.

\begin{align*}&\mathbf{L}_{\mathrm{ij}}[\mathrm{pH}]=\\ &\qquad\qquad~~\begin{array}{ccccc}Rung_1 & \ldots & Rung_8 &\hspace{-0.2pc} \ldots & Rung_{16}\end{array}\\ &\hspace{0.5pc}\begin{array}{c}Rung_1 \\\ldots \\ Rung_8 \\ \ldots \\ Rung_{16}\end{array}\left(\begin{array}{ccccc} 3.57 & \ldots & -0.09 & \ldots & 0.09 \\ \vdots & \vdots & \vdots & \vdots & \vdots \\ -0.09 & \ldots & 3.57 & \ldots & -0.09 \\ \vdots & \vdots & \vdots & \vdots & \vdots \\ 0.09 & \ldots & -0.09 & \ldots & 3.57 \end{array}\right) \end{align*}

**FIGURE 2.**
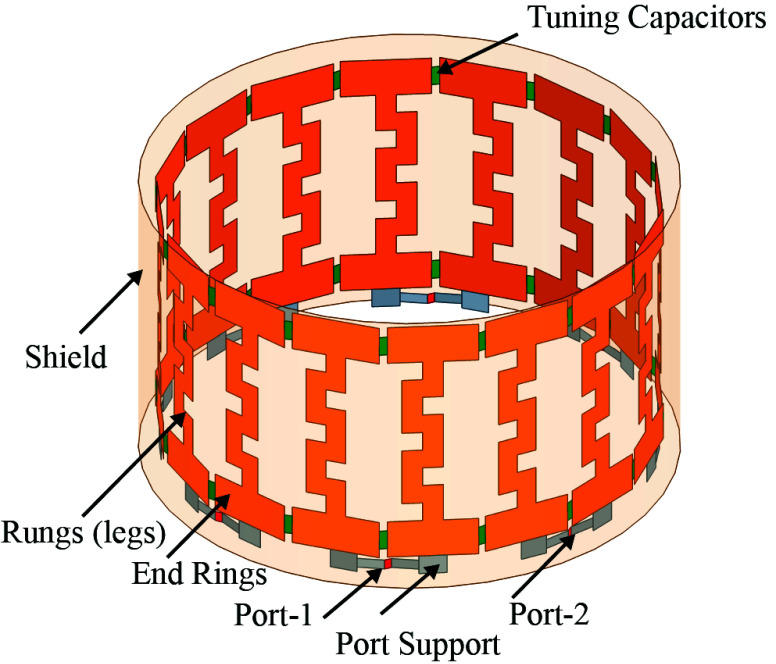
The proposed Birdcage coil in high-pass configuration includes 16 meander line coils and 32 tuning capacitors, with eight ports positioned at a 45° displacement from each other.

These inductances correspond to the circuit shown in Figure 4, where $L_{er}$ denotes the end-ring inductance and $L_{r1}$ represents the rung inductance. Since there are 16 rungs (as shown in Figure 2), the matrix must be $16 \times 16$. This matrix is essential for determining the values of the tuning capacitors needed to achieve a specific resonance frequency by solving the circuit in Figure 4.

**FIGURE 3.**
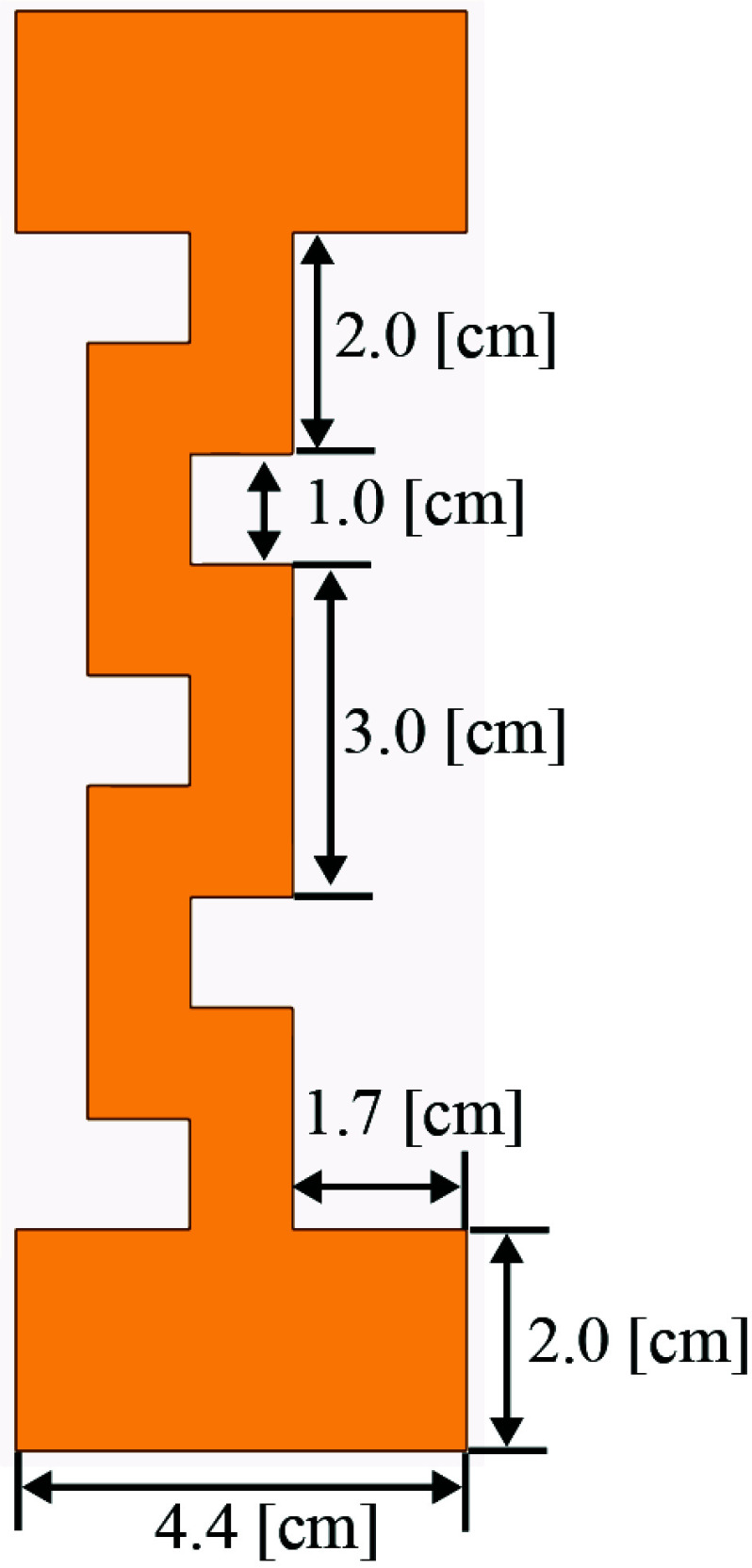
The proposed Birdcage based meander line a single coil with dimensions in [*cm*].

**FIGURE 4.**
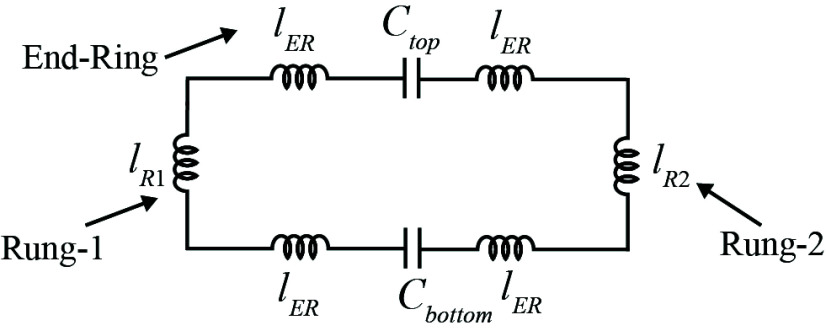
The equivalent circuit of the proposed birdcage based meander line antenna.

This article introduces an innovative Birdcage coil design that incorporates meander line antennas. Figure 2 illustrates the configuration of the proposed coil, which comprises eight ports and utilizes 32 tuning capacitors arranged in a high-pass configuration. Notably, each of the 16 rungs forming the coil is designed as a meander line antenna. Detailed dimensions of these rungs are provided in Figure 3, offering specific insights into their structure and dimensions. The straight length of this coil is 13 cm including the end rings on the top and bottom of the antenna. The antenna’s RLCG circuit, represented in Figure 4, is analyzed using Ansys Q3D Extractor. This software computes matrix entries by individually exciting conductor nets or defined source terminals. Each excitation generates column entries in the matrix, calculating capacitance, inductance, or resistance for the specific conductor concerning the reference ground and other conductors [39]. The resistance and inductance matrices contain rows corresponding to each source terminal [39]. Meanwhile, the capacitance and conductance matrices have rows representing individual nets, illustrating the precise electromagnetic interactions between components in the antenna setup. In Figure 4 $l_{R}$ refers to the equivalent inductance of the antenna’s rung, and $l_{ER}$ is the equivalent inductance of the end rings of the antenna. Inductance *L* directly correlates to the energy stored within a magnetic field and it is proportional to the energy stored $E_{s}$ in the magnetic field when current *i* flows.

\begin{equation*} E_{s}=\frac {1}{2}Li^{2} \tag {1}\end{equation*}

To calculate inductance using Ansys Q3D Extractor a current of 1 ampere is directed through a single conductor, while no current is permitted to flow through any other conductor. Then the energy contained within the magnetic field, specifically associated with the inductance between two conductors, is mathematically expressed by the following relationship:

\begin{equation*} E_{s_{ij}}=\frac {1}{2}\int (B_{i}H_{j})dl \tag {2}\end{equation*}

The equation defines $E_{s_{ij}}$ as the energy stored within the magnetic field connecting rung *i* to rung *j*. $B_{i}$ represents the magnetic flux density and $H_{i}$ denotes the magnetic field intensity. This relationship establishes the stored energy within the magnetic field, specifically capturing the interaction between these designated conductors in the system. The complete inductance matrix in $(pH)$ is given above. The diagonal entries of this matrix signify the self-inductance of each rung, whereas the off-diagonal elements indicate the mutual inductances between the rungs. The positive and negative signs in the off-diagonal elements of the matrix signify additive or subtractive coupling between the rungs.

**FIGURE 5.**
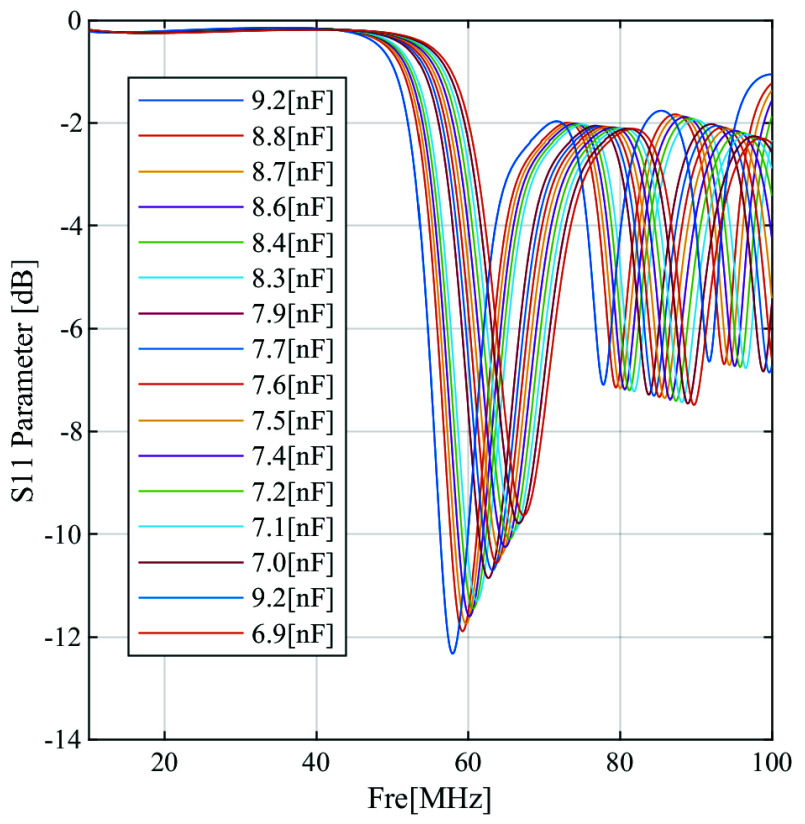
The $S_{11}$ parameter in (dB) at different tuning capacitors in $(nF)$.

**FIGURE 6.**
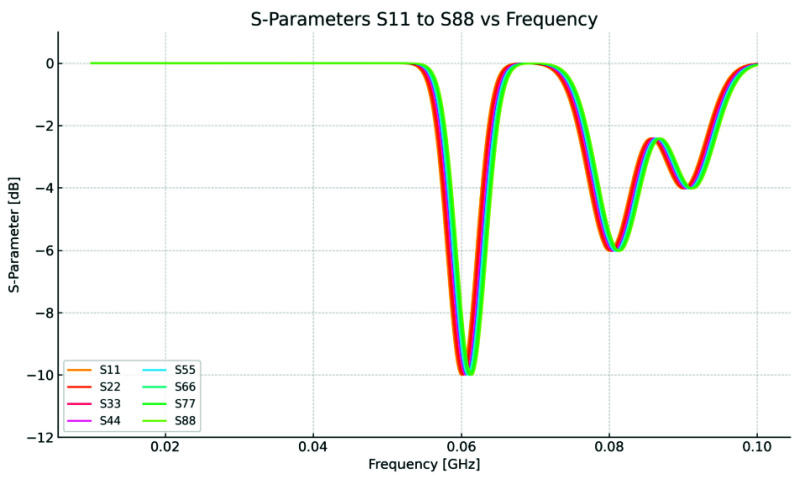
The $S_{ii}$ parameter in (dB) (from top to bottom): $S_{11}$, $S_{22}$, $S_{33}$, $S_{44}$, $S_{55}$, $S_{66}$, $S_{77}$, $S_{88}$. The $S_{ii}$ parameter was measured with the human head model in place. This should help to better illustrate the antenna’s behavior in a loaded state, which is a key factor for evaluating its real-world performance.

The two capacitors, denoted as $C_{top}$ and $C_{bottom}$ serve as external tuning components essential for fine-tuning the antenna’s frequency response. The determination of their specific values involves iterative simulations via Ansys Circuit simulation. The objective is to adjust $C_{top}$ and $C_{bottom}$ iteratively until the antenna’s response frequency aligns precisely with the targeted frequency of 64 MHz. This precise process ensures that the antenna operates optimally at the desired frequency, achieving the desired performance characteristics. The presence of a load (human body model) on the coil can cause a shift in the resonant frequency due to coupling between the coil and the loaded models. Consequently, it is essential to repeat the tuning procedure to readjust the resonant frequency back to 64 MHz. Ansys Circuit package was employed for antenna tuning, exploring various capacitor values as depicted in Figure 5. Using 32 capacitors on both top and bottom yielded equal values of 75.33 [pF] each, precisely attaining resonance at 64 MHz. Figure 6 depicts the *S* parameter in decibels $(dB)$ for all the eight ports, clearly demonstrating resonance of the proposed design at 64 [MHz]. The $S_{ii}$ parameter was measured with the human head model in place. This should help to better illustrate the antenna’s behavior in a loaded state, which is a key factor for evaluating its real-world performance. The proposed numerical antenna accepted power $P_{acc}$ is simulated and found to be 124 [W] to produce the SAR of 0.4-0.9 W/kg.

## VI. Human Body and Brain Model

The human body adopted in this article is the NEVA human body simulation model. The NEVA-Female v.3.0, is a platform-independent full-body computational human model, is constructed from 26 distinct tissues and 233 separate tissue parts [40], [41]. These components are presented as 3D CAD objects in the form of triangular surface meshes, totaling around 160,000 facets. Its accuracy is marked by a surface deviation error of 0.5-3 mm within the cranium and up to 7 mm across the main body, ensuring a highly detailed representation of human anatomy [41].

**FIGURE 7.**
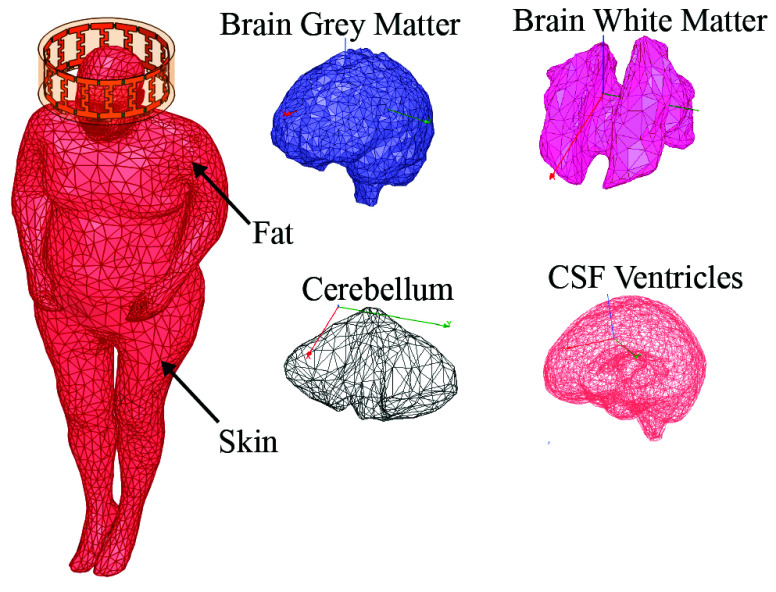
The NEVA human body model incorporates the proposed antenna affixed to the head.

**FIGURE 8.**
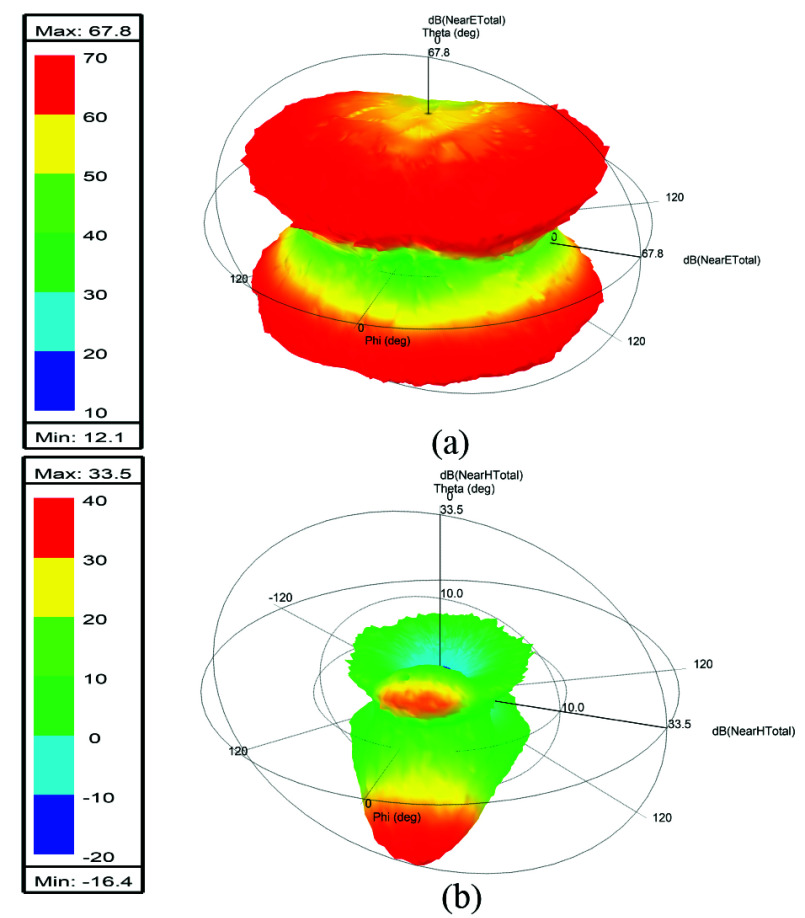
(a) Near electric field in (dB). (b) Near magnetic field in (dB) of the proposed design.

The human body parts included in this study are the following: 1) trachea sinus. 2) Arteries and upper veins. 3) Skin. 4) Bone cortical. 5) Brain white matter. 6) Brain grey matter. 7) Cerebellum. 8) Cerebrospinal fluid (CSF Ventricles, and CSF extra and outer shells). 9) Eye vitreous humor for both left and right eyes. 10) Body fat (averaged). TABLE 1 presents the dielectric properties of various tissues at a specific frequency of 64 [MHz]. These dielectric parameters have been determined based on the Gabriel dispersion relationships [42]. Figure 7 illustrates the NEVA human body model, highlighting the primary components of the brain. Figure 8(a) and 8(b) display the near electric and magnetic fields of the proposed 8-ports antenna, respectively.

**TABLE 1:** Dielectric Properties of Human Body Parts found at 64 MHz [42].

Tissue	Relative Permittivity	Electrical Conductivity(S/m)
trachea sinus (air)	1.00E+0	0.00E+0
Arteries (blood)	8.64E+1	1.21E+0
Skin	9.22E+1	4.36E-1
Bone cortical	1.67E+1	5.95E-2
Brain white matter	6.78E+1	2.92E-1
Brain grey matter	9.74E+1	5.11E-1
Cerebellum	1.16E+2	7.19E-1
Cerebrospinal fluid	9.73E+1	2.07E+0
Eye vitreous humor	6.91E+1	1.50E+0
Body Fat	1.36E+1	6.62E-2

## VII. Results and Discussion

Specific Absorption Rate (SAR) refers to the rate at which the human body absorbs electromagnetic energy when exposed to radio frequency waves. It’s given in watts per kilogram ( $W/Kg$) and serves as a crucial metric in evaluating potential health implications related to radio frequency exposure from various devices and antennas. More details about specific absorption rate (SAR) is given in the Appendix. SAR values help establish safety thresholds and guidelines to mitigate potential health risks associated with electromagnetic radiation absorption in human tissues and can be calculated using equation (3), following the research parameters for AZ treatment invented at the IUSM, where $\sigma $ is the electrical conductivity [S/m], and $\rho $ is the tissues density in $[kg/m^{2}]$, and E is the electric field intensity in [V/m] [43].To ensure a Specific Absorption Rate (SAR) of 0.4 W/kg and 0.6 W/kg, the maximum required magnetic field strengths is 0.001 Tesla under loaded condition as illustrated in Figure 23.

\begin{equation*} SAR = \int _{V} \frac {\sigma }{\rho } |E|^{2} dV \tag {3}\end{equation*}

**FIGURE 9.**
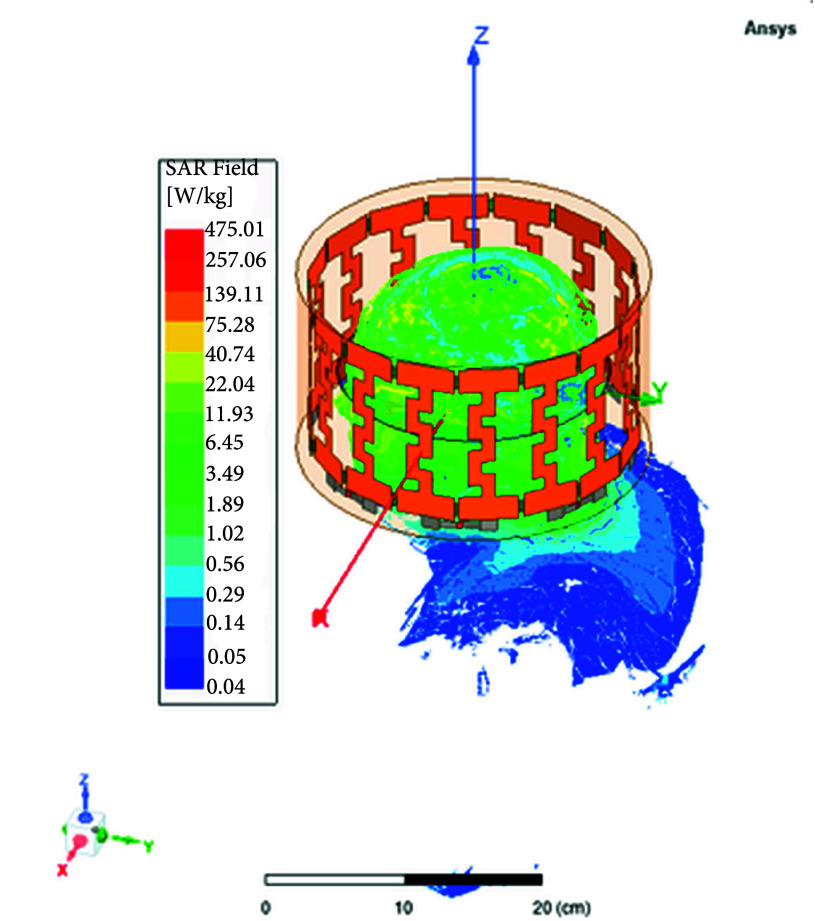
Average SAR distribution across the brain in [ $W/Kg$] using the 8-port antenna.

**FIGURE 10.**
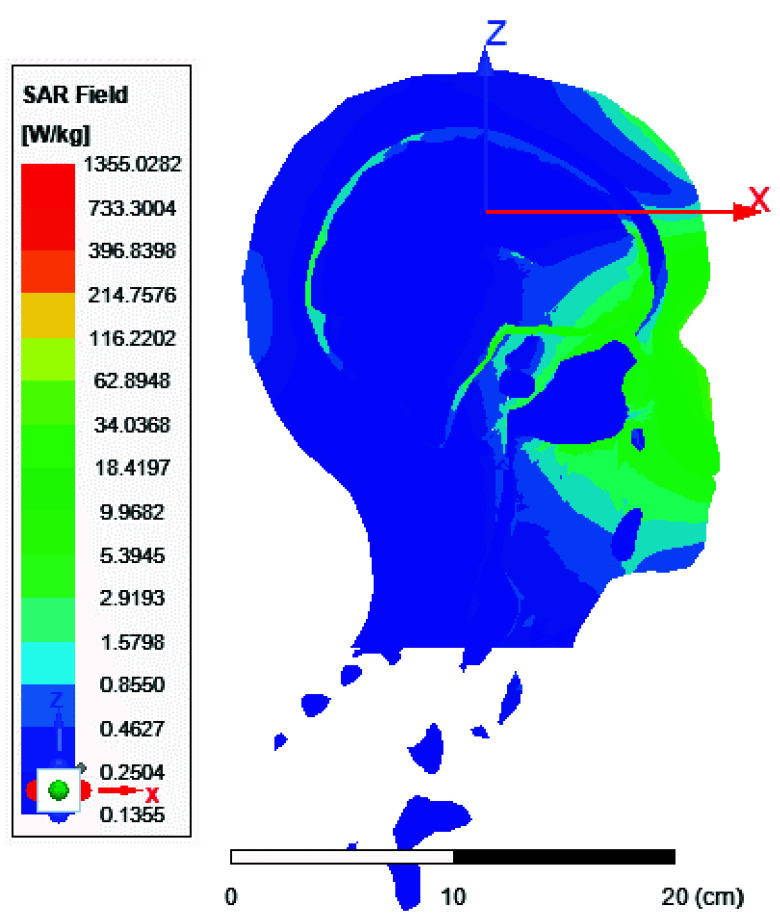
Local SAR distribution across the brain in [ $W/Kg$] using the 8-port antenna (xz plane view).

**FIGURE 11.**
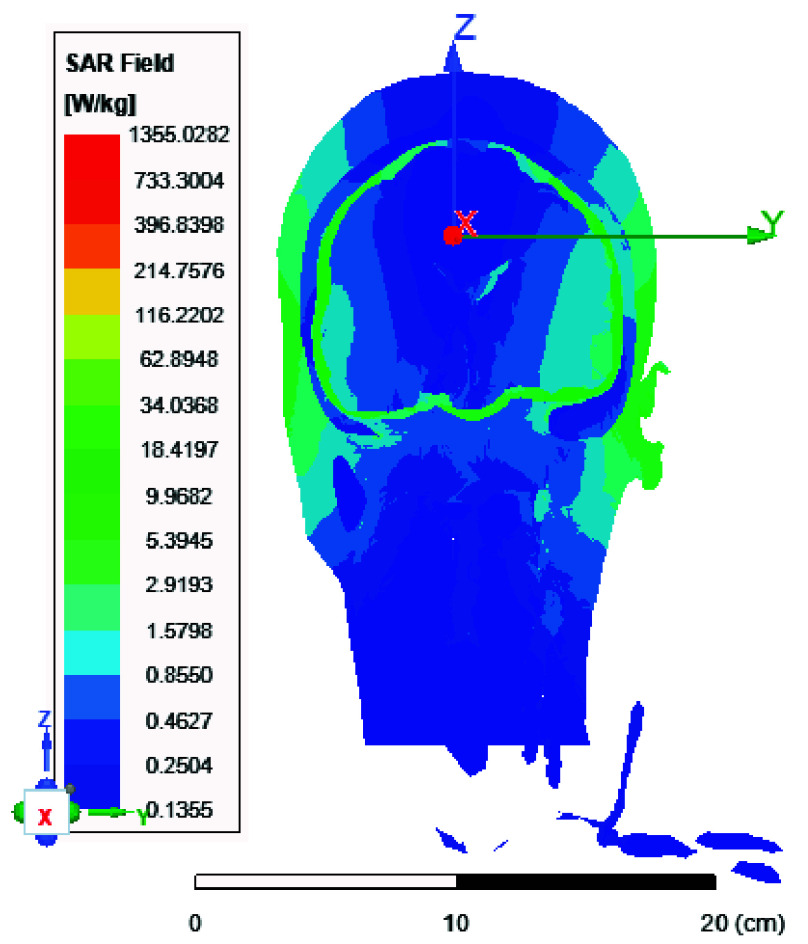
Local SAR distribution across the brain in [ $W/Kg$] using the 8-port antenna (yz plane view).

**FIGURE 12.**
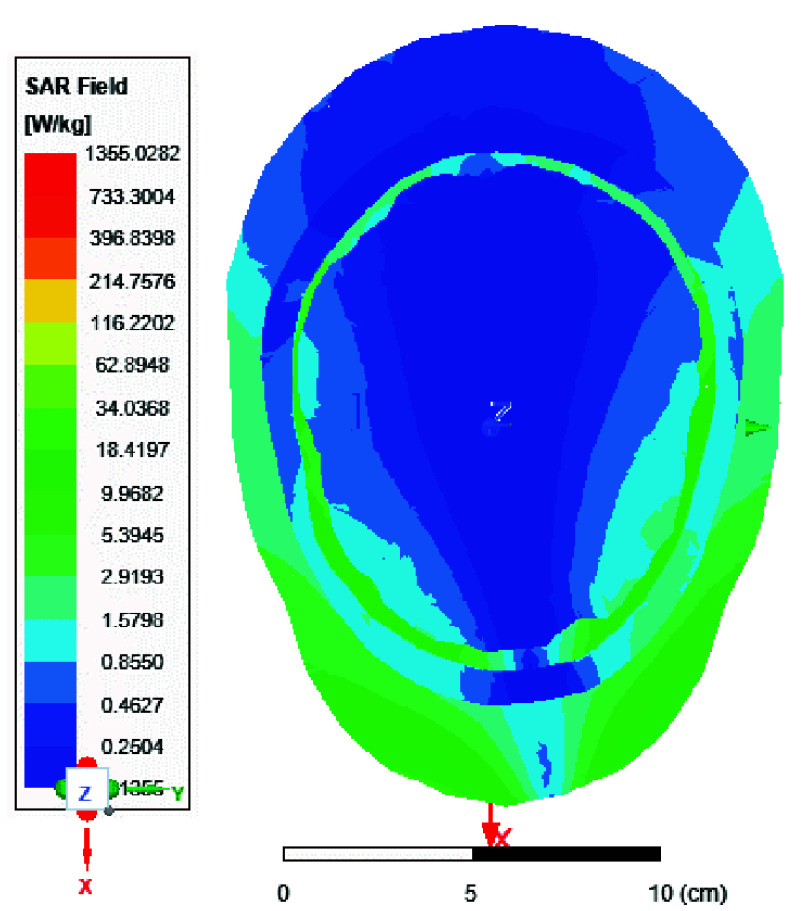
Local SAR distribution across the brain in [ $W/Kg$] using the 8-port antenna (xy plane view).

**FIGURE 13.**
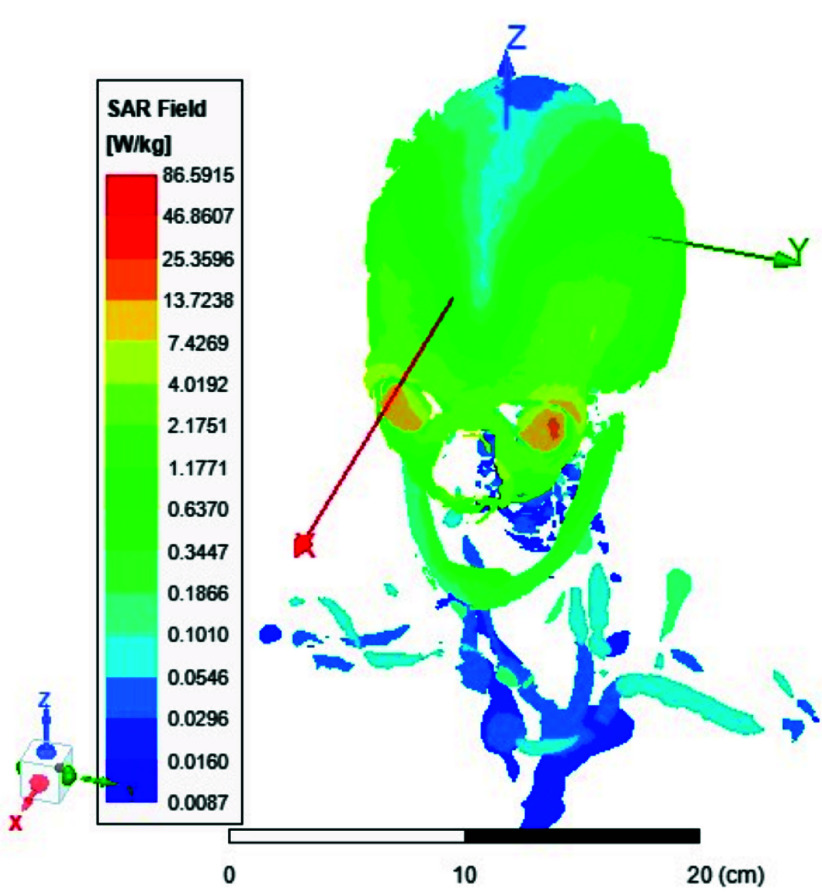
Average SAR [W/kg] for the two-ports antenna.

**FIGURE 14.**
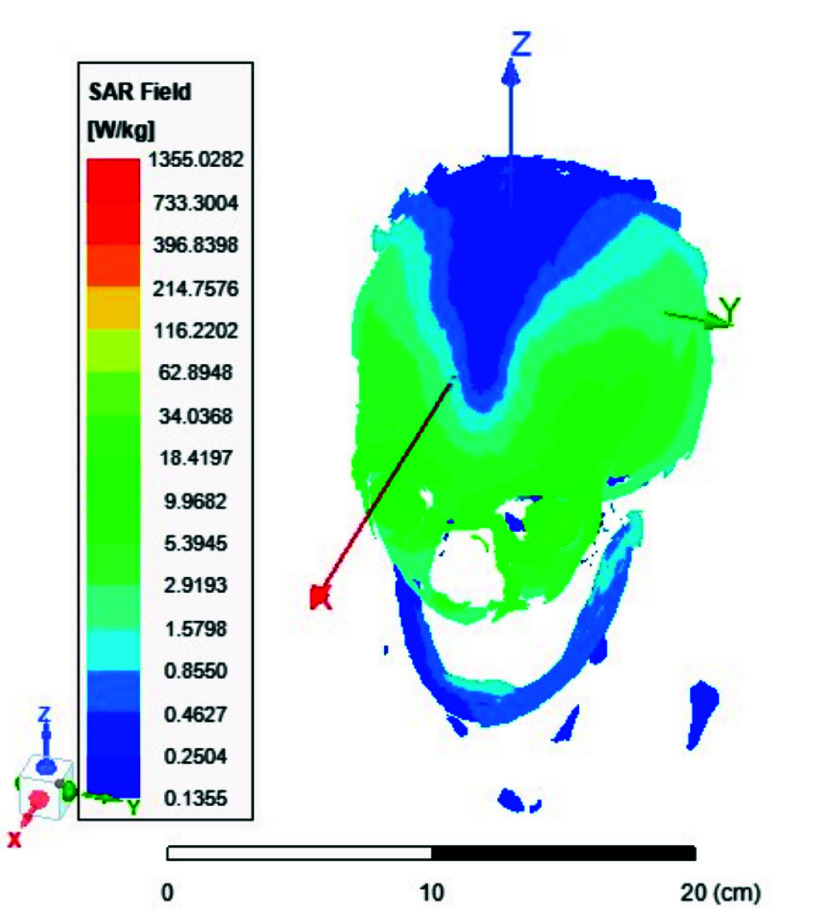
Average SAR [W/kg] for the eight-ports antenna.

**FIGURE 15.**
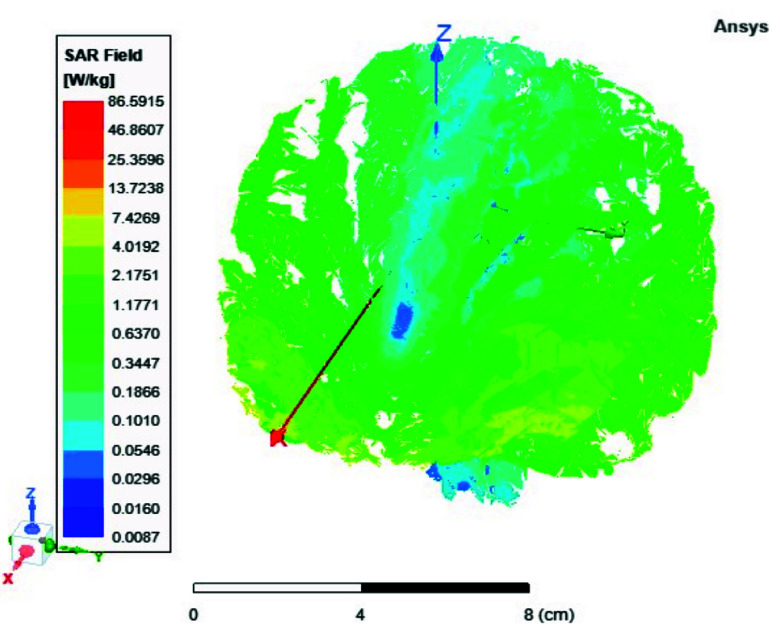
Local SAR [W/kg] recorded at brain grey matter tissues for the two-port antenna.

**FIGURE 16.**
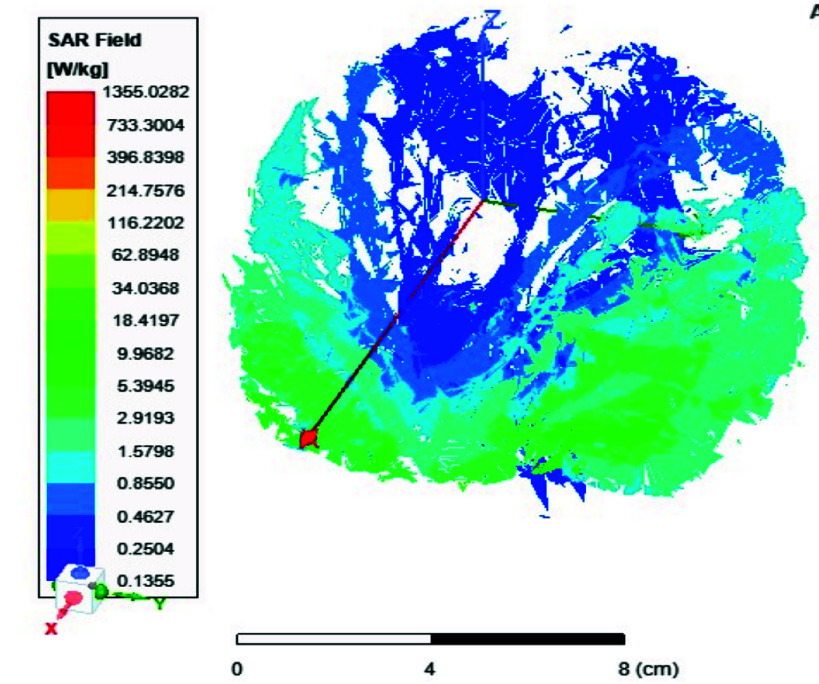
Local SAR [W/kg] found at brain grey matter tissues using eight ports antenna.

**FIGURE 17.**
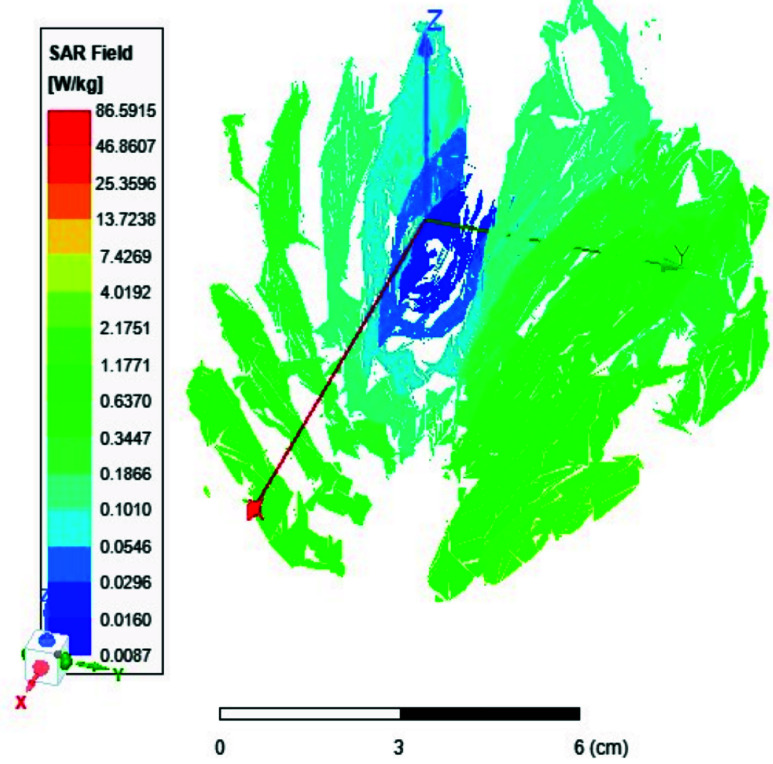
Local SAR measured at the brain white matter for two-port antenna.

**FIGURE 18.**
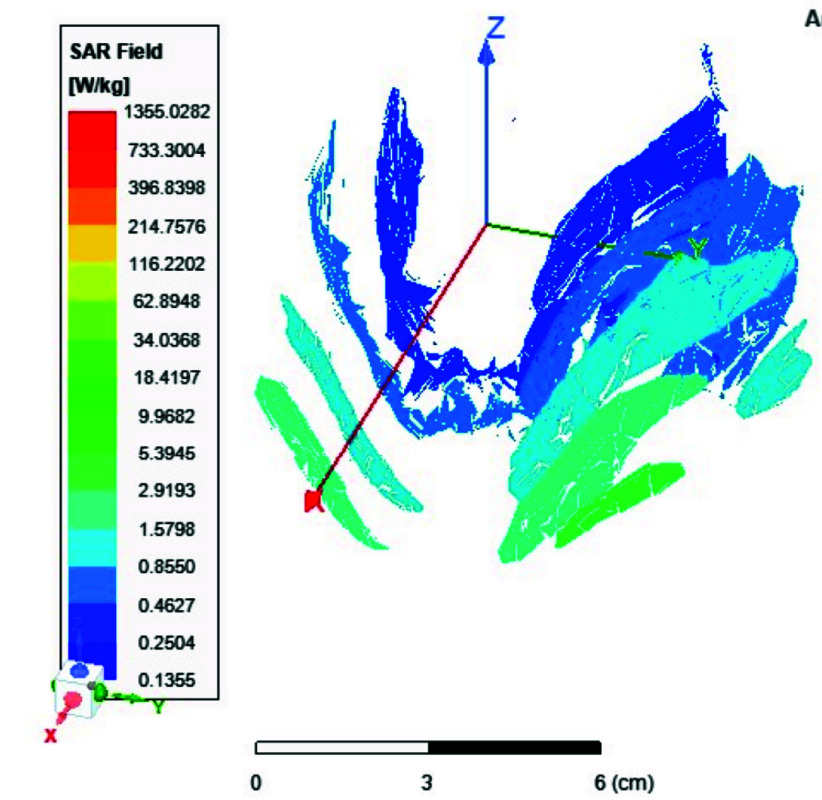
SAR measured at the brain white matter for eight-port antenna.

**FIGURE 19.**
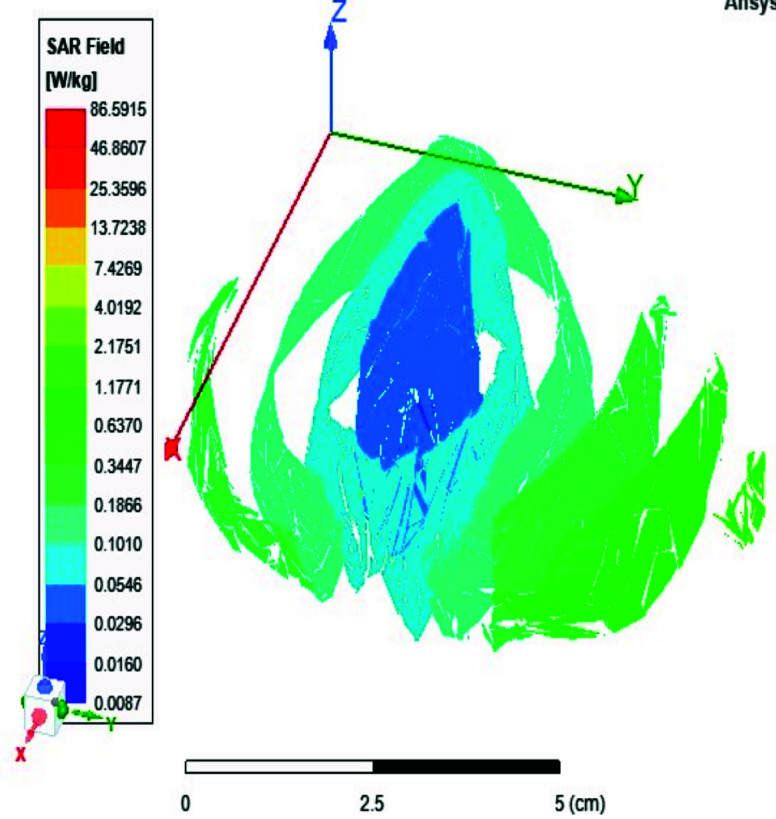
Local SAR at Cerebellum tissues for two-ports case.

**FIGURE 20.**
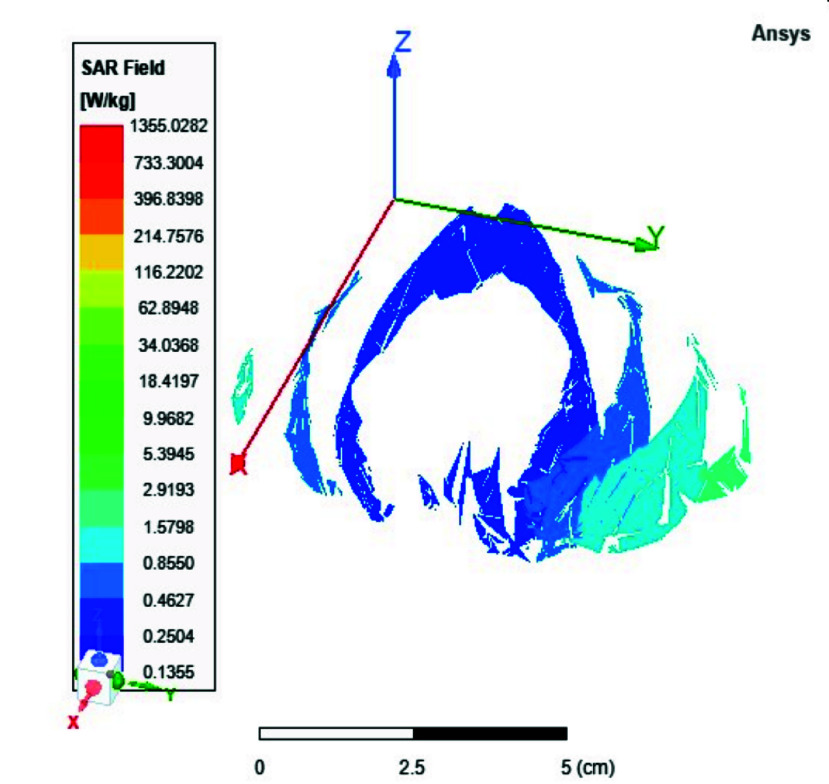
Local SAR at Cerebellum tissues for eight-ports case.

**FIGURE 21.**
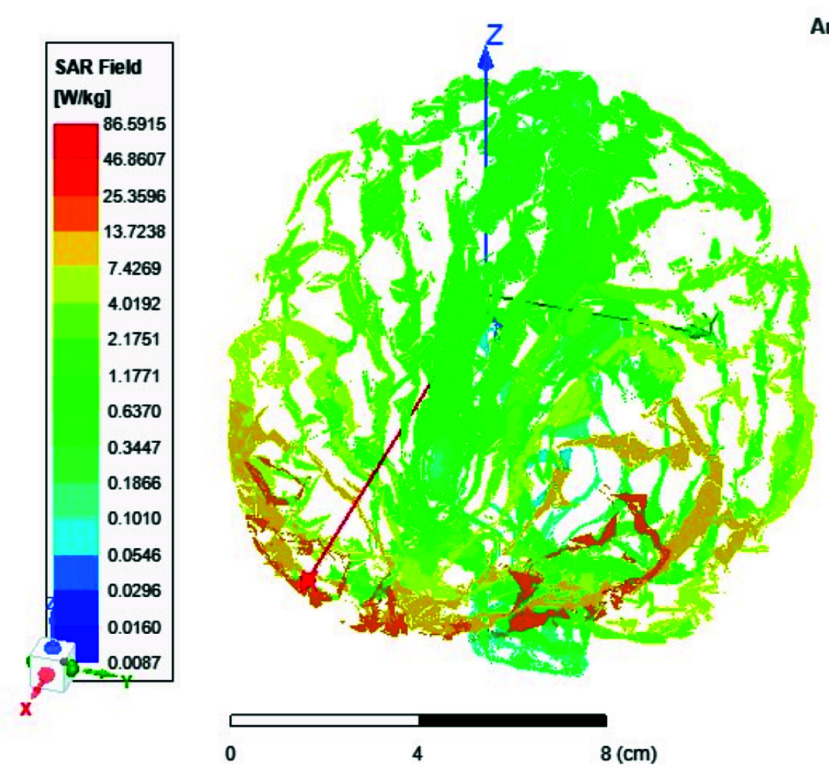
Local SAR at the CSF shell using two port case.

**FIGURE 22.**
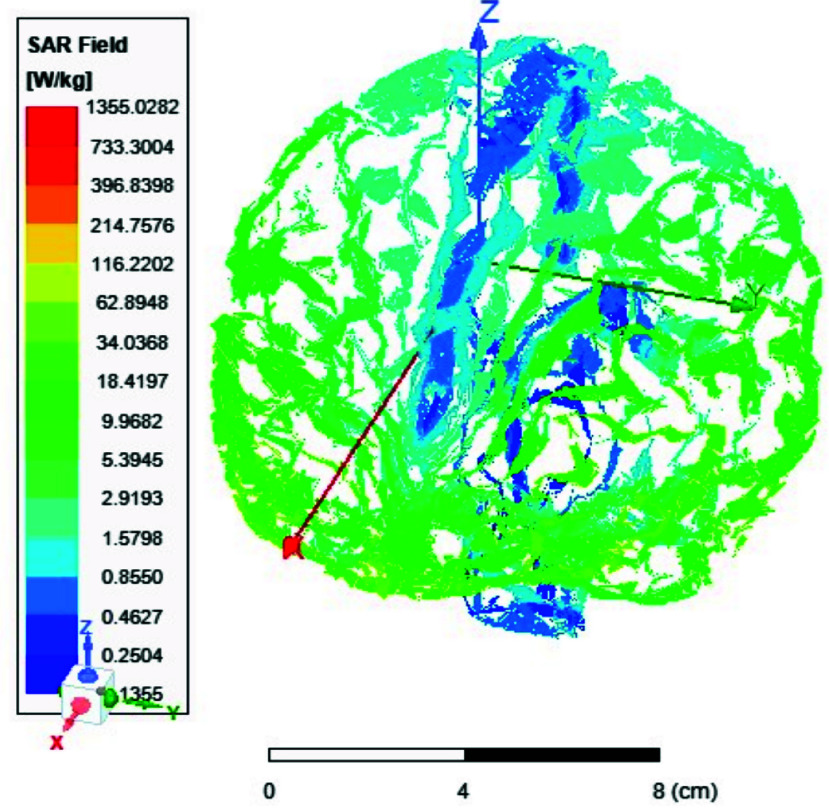
Local SAR at the CSF shell using eight ports case.

**FIGURE 23.**
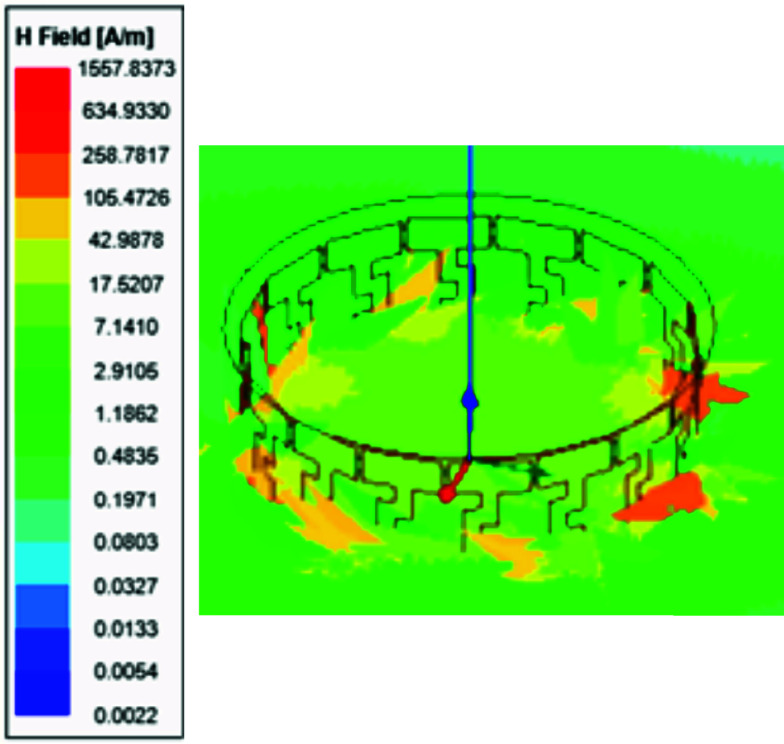
To ensure a Specific Absorption Rate (SAR) of 0.4 W/kg and 0.6W/kg, the maximum required magnetic field strengths is 0.001 Tesla underloaded condition.

In this section, we delve into the examination of both local and average Specific Absorption Rate (SAR) in accordance with the standards outlined in IEC/IEEE 62704-4. The averaging process is conducted across a mass of 1 gram of tissue. The average SAR values ranging from 0.4 to 0.9 W/kg achieved within the simulated brain tissues using the proposed 8-ports antenna, as depicted in Figure 9, comply with safety guidelines by FDA. The local SAR for 1 gm of tissues was assessed across various brain regions and documented from different cross-sectional planes. First, Figure 10 displays the spatial distribution of specific absorption rate (SAR) in [W/kg] as observed from the xz-plane. Second, Figure 11 depicts the local specific absorption rate (SAR) in [W/kg] as observed from the yz-plane. The third representation in Figure 12 illustrates the local specific absorption rate (SAR) observed within the xy-plane.

## VIII. Comparison Between the Proposed Antenna with Two and Eight Ports Designs

Local and average Specific Absorption Rate (SAR) are investigated in this section. To achieve the most homogeneous (SAR),the proposed antenna models were excited with two distinct phase shifts between the RF signals at the feed ports: 1) Coil with two ports at a phase shift of 90°, and 2) Coil with eight ports excited at 45°. The average and local Specific Absorption Rate (SAR) in [W/kg] were simulated and recorded at various regions of the human body for both configurations: the two-port and eight-port setups, the results are illustrated in Figure 13 to Figure 22. Figure 13 shows the average SAR [W/kg] for the 90° phase shift. while Figure 14 illustrates the average SAR [W/kg] for the eight-ports antenna. Figure 15 depicts the local SAR [W/kg] simulated at brain grey matter tissues for the two-port antenna and Figure 16 shows the local SAR [W/kg] recorded at brain grey matter tissues using eight ports antenna. Figure 17 illustrates the local SAR simulated and found at the brain white matter tissues using the two-port antenna. Figure 18 shows the local SAR at the brain white matter tissues using the eight-port antenna. While Figure 19 depects the Local SAR at Cerebellum tissues using the two-ports case and Figure 20 shows the local SAR at Cerebellum tissues using the eight-ports case. Lastly Figure 21 illustrates the local SAR at the CSF shell using two port case and Figure 22 is the local SAR at the CSF shell using eight ports antenna. The eight-port coil demonstrates improved SAR homogeneity, maintaining values ranging from 0.6 to 0.9 [W/kg] within brain tissues compared to the two-ports case.

## IX. Temperature Estimation Based on the Proposed MRI Coil

**FIGURE 24.**
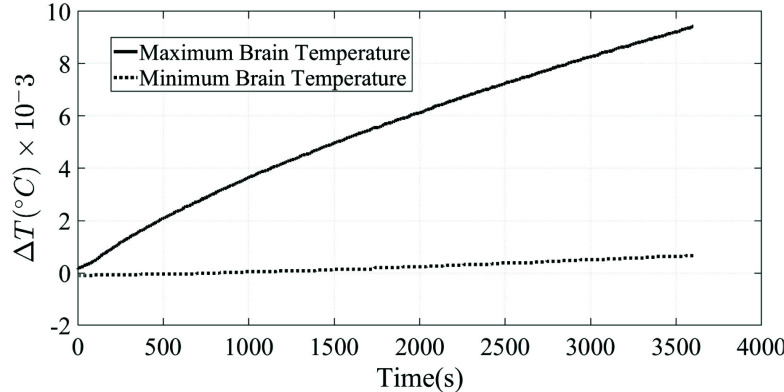
Maximum and minimum brain temperature.

In order to further comply with safety guidelines by the FDA, the thermal response of the human body model during the exposure to electromagnetic waves from the 8-port antenna was obtained using finite-element analysis. The starting temperature of the human-body model and the ambient temperature were 37 degrees Celsius, and then the fields inducing average SAR between 0.4 and 0.8 W/kg were applied and the transient thermal response calculated for 3600 seconds. Figure 24 shows the maximum temperature rise (0.01 degree Celsius) in the simulated brain after one hour of RF power deposition with a SAR of 0.4-0.9 Wkg generated by 100 Watt of input power.

Previous research has investigated the relationship in humans between RF exposure, and the resulting SAR and temperature elevation, with the goal of monitoring the temperature increase to remain within human safety guidelines. With out proposed novel birdcage coil operating at 64 MHz with the goal of 0.6 W/Kg SAR throughout the human head, other research can be examined for a baseline of where the temperature rise for such a device might lie. Christopher M., et al concluded that there isn’t a straightforward relationship between SAR and temperature increase within human issues, and found through calculation using the FDTD method, that a birdcage coil at 64 MHz achieving a head average SAR of 3.0 W/kg resulted in a maximum 2.1 degree Celsius increase in the head, and maximum 0.9 degree Celsius increase in the brain. Reference [44] Collins et al. also using numerical methods, found that when using birdcage coils from 64 to 400 MHz causing a head average SAR of 3.0W/kg, that a maximum temperature increase of 0.87 degrees Celsius occurred in the brain [45]. They had also concluded however that the relationship between SAR and temperature increase is not straightforward and should be better understood. Reference [45] Hirata et al. used numerical simulations on male and female human body models, using various models with different number of tissues, and exposing them to a 123 MHz 16 rung coil until a whole body averaged SAR of 2 W/kg was reached [46]. Under these conditions, the full male body model reached a head average SAR of 3.58 W/kg and an average head temperature increase of 1.05 degrees Celsius, while the female model reached a head-average sar of 2.25 W/kg with an average head temperature increase of 0.72 degrees Celsius [46]. Also using FDTD methods for 1 hour simulations of RF exposure for male and female body models, Hironori Sugiyama, et al found ¡1 degree core-temperature increase for the body as a whole, when at 4 W/kg whole-body average SAR [45]. They had concluded that estimates for a temperature and SAR relationship could be made using a proposed novel formula when considering whole-body average SAR and core-temperature, however for our usage a relationship for more granular information would be necessary [45]. Finally, in a study using a dipole antenna operating in the range between 800MHz and 3GHz and using FDTD numerical methods, Hirata et al. found a less than 0.1 degree Celsius temperature increase within brain tissue for 10g average SAR of less than 1 W/kg [47]. It was also found that there was difficulty in establishing a consistent SAR and temperature elevation relationship across different tissue types, SAR measurements and operating frequencies [47]. Since the thermal response is considered an important element towards the system stability, the NEVA head model was simulated via HFSS with seven layers of the brain tissue. The simulation run for an hour to track the temperature change with the 0.6 SAR power. The change in milli kelvin suggests good stability and flexibility for patient therapy in medical settings.

## X. Limitations and Future Work

The future direction of this work lies in taking the success seen in numerous preclinical studies of cell cultures, AD animal models, and one clinical trial and applying it to human clinical scenarios. The challenge comes in the translation of these results to human characteristics. Because we cannot transpose the RF power deposition and frequency in cell cultures and animal models 1:1 to human studies, our objective was to find the frequency and input power of the RF source that would generate an RF power deposition with a specific absorption rate (SAR) of 0.4–0.9 W/kg in a numerical human brain phantom using mathematical models, computer simulations, and practical validation experiments considering the different human head tissues and their electrical properties. To determine the cause or functional dependence of RF-induced biological effects, regulatory agencies have recommended measuring the energy absorbed or power deposition with a SAR value by tissues and cells.

### A. Sar Use

SAR is an important safety guardrail for humans when using RF exposures. The biological effects of RF exposures cannot be determined by measuring power density unless the amount of energy absorbed is also known [48], [49]. SAR measures the rate of the energy absorbed or power deposition in the tissues relevant to specific biological effects and is, therefore, a central consideration for the envisioned work. Given that we found that a power deposition with a SAR of 0.4–0.9 W/kg lowers A $\beta $ levels in mice and human brain cultures, we must determine the EMF parameters of the external RF source that will produce the RF power deposition with a SAR of 0.4–0.9 W/kg in a numerical human head with all its tissue layers before we use it in humans. SAR determination will be obtained by numerical field calculations with suitable coils and patient models using the finite-difference time-domain (FDTD) technique and adjusting the EMF parameters of the external RF field source to produce an RF power deposition with a SAR of 0.4–0.9 W/kg and to allow visualization of a homogeneous power distribution.

### B. Sar Framework

To find a SAR framework, we reviewed the negative or positive actions of REMFS treatments on memory [50] and in AD pathology in multiple studies. For this purpose, we used the inverted U-shaped dose-effect curve (IUSDEC). Initially, we reviewed the literature [15], [51], [52] from cell culture [16], [53], [54], [55], [56], [57], [58], [59], animal [13], [17], [20], [21], [22], [23], [24], [25], [26], [27], [28], [30], [60], [61], [62], [63], and human [64], [65], [66], [67], [68] studies before we performed our human brain culture studies. We found that an RF power deposition that results in a SAR between 0.25–5 W/kg improves AD pathology and memory. On the contrary, when the SAR was lower than 0.25 W/kg [69], [70], [71], [72], [73], [74], [75], [76], [77], [78], [79], [80], [81], [82], [83] or higher than 5 W/kg [84], [85], [86], [87], [88], [89], [90], [91], [92], [93], [94], [95], [96], [97], [98], it had no effects or was detrimental to AD pathology and cognition, suggesting an IUSDEC.

### C. Frequency Range

The penetration depth of EM radiation in tissues increases as frequency is reduced [99]. Thus, RF below 400 MHz is best suited for human exposure to tissues lying 1–20 cm deep in the body, such as the head. We chose 64 MHz for our studies for several reasons:

1) Ideal penetration depth (13.5 cm) [100] and homogeneous field distribution.
2) Previous studies on human cells and mouse cultures did not find toxicity [101].
3) It is in the range 30–200 MHz [102] at whole human body resonance, so less power is needed to obtain SAR with a lower temperature rise (TR) [103].
4) It has been used by MRI systems for 40 years, thus, providing an established and safe framework for human exposure.

### D. Future Experimental Validation

**FIGURE 25.**
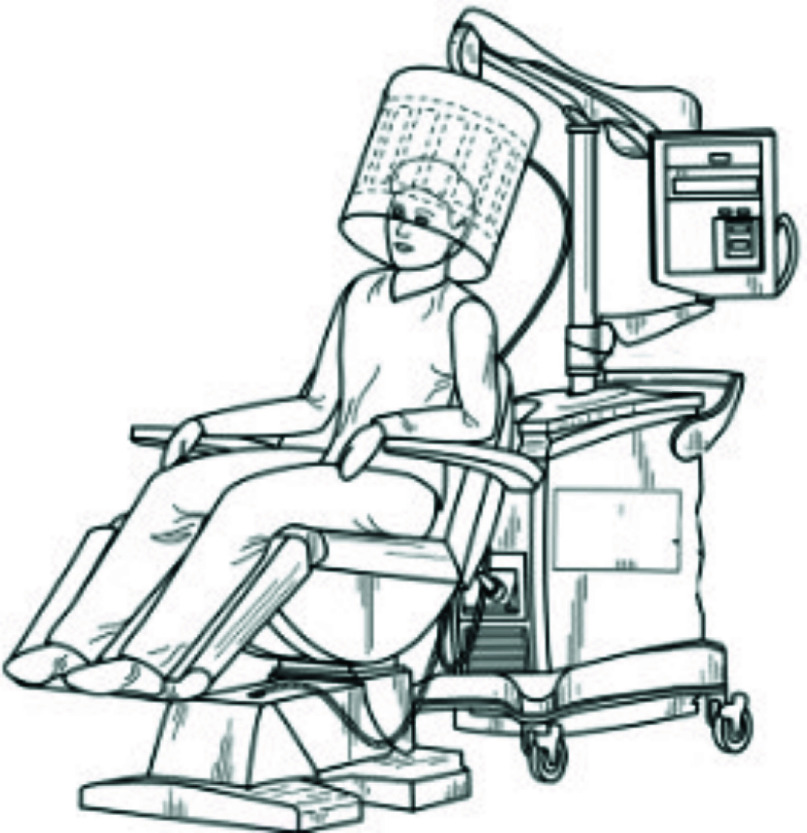
Perspective view of an EMF generation system (REMFS) with a head-mounted antenna, including control system and coupling unit.

This paper focuses on the design of an RF device appropriate for human use. After we optimize the EMF parameters for safe and effective human exposure in computer simulations, we will manufacture the prototype. Figure 25 shows a Perspective view of an EMF generation system (REMFS) with a head-mounted antenna, including control system and coupling unit. We will validate the device and the parameters obtained in our simulation, such as input power, output power, distance from the antenna, the number of excitation ports, and phase shift. We will test our prototype on a specific anthropomorphic mannequin (SAM) [104], which accurately reproduces the thermal and SAR effects of a human head under electromagnetic exposures. The SAM phantom will have tissue-simulating liquids that represent a multi-layered, multi-tissue, and multi-organ head phantom in an anatomical form using a wide range of tissue-simulating media at an extended frequency range at approximately 64 MHz. We will expose the SAM in our laboratory with the EMF parameters found in our simulations for 120 minutes, considering the plan of subsequently testing the approach in future human studies. We will use an EM Dosimetric Assessment System (DASY) [105] with multiple SAR sensors to map its distribution. We will also use a Thermometry System [106] using multiple probes where our simulation results show the maximum temperature rise (TR). We will maintain the TR to <0.5° C by negative feedback control, adjusting the power of individual ports adjacent to the temperature increase. After we validate our device and the EMF parameters, we plan to test for efficacy and safety initially in “first-in-human testing”, then, we will perform a Phase I clinical trial in AD patients with Mild Cognitive Impairment (MCI).

### E. Clinical Settings

A future general implementation strategy to integrate REMFS into existing AD treatment workflows will consist of the following: 1) The inclusion criteria will be patients with Amnestic mild cognitive impairment due to AD diagnosed by Neuropsychological testing. 2) The required diagnostic tests will be Brain MRI, FTDG PET scan, and Amyloid PET to determine the brain ( $A\beta $) load and the degree of neurodegeneration. 3) The treatment plan will include daily REMFS exposures in combination with Cholinesterase inhibitors and Memantine. 4) Treatment administration will ensure the correct RF power deposition with a SAR of 0.4-0.9 W/kg daily for one hour, provided by a birdcage antenna. 5) During the exposure, SAR and temperature will be monitored and controlled. 6) The follow-up care will include appointments, medication refills, and lifestyle recommendations. The REMFS portable size will allow us to provide treatments to patients at hospitals, neurology clinics, psychiatry clinics, geriatric clinics, nursing homes, and out-of-pocket home consumers in the USA and later in the world. In the clinical setting, another area of interest lies in how to determine the real-time effectiveness of REMFS for patients. New blood and spinal fluid biomarkers such as phosphorylated tau 217 (p-tau 217) and A $\beta _{42}$:A $\beta _{40}$ hold promise as future markers that can be monitored pre and post-REMFS treatment. Temperature monitoring remains an utmost safety concern as well. Since RF heat can cause tissue injury, it must be kept to a safe level of less than 0.5°C per regulatory agencies [31]. Before investigating human trials, there must be clearly defined parameters for a safe SAR level that does not exceed this threshold in all patients. AI deep learning (convolutional neural networks) holds potential for simulating personalized treatment with the appropriate temperature and SAR levels while accounting for unique patient characteristics based on the patient’s MRI sequences. Preclinical data suggest that the optimal treatment time is one hour once a day of RF exposures, and clinical trials also need to determine the exposure time and schedule. Given the circadian production and clearance of amyloid- $\beta $ (A $\beta $) [107], especially early in the disease course, this might affect the duration and timing of treatment.

While the birdcage-based MLA design demonstrated improved SAR homogeneity in simulations, future clinical studies and phantom testing are required to verify uniformity under real physiological loading conditions. Integration into current Alzheimer’s care will be supported through modular use with existing MRI tools, as well as biomarker monitoring frameworks. The SAR levels of 0.4-0.9 W/kg at 64 MHz are comparable to routine MRI scans, which have been safely used in patients for over four decades. Additionally, minimal $(< 0.5^{\circ })$ temperature elevation in simulated tissue during one-hour exposures.

Compared to Transcranial Magnetic Stimulation (TMS), which primarily affects superficial cortical areas, and transcranial photobiomodulation (tPBM), which suffers from limited penetration depth due to light scattering, REMFS delivers uniform energy to deep brain regions. The selected 64 MHz frequency yields a skin depth of approximately 13.5 cm in brain tissue, reaching key memory regions such as the hippocampus. Furthermore, SAR levels can be dynamically regulated in real-time, providing a tunable and safer alternative to more invasive approaches [108].

Another REMFS advantage is that it showed minimal long-term side effects in preclinical studies and clinical trials. A REMFS clinical trial at 915 MHz only caused mild discomfort after two years and a half of RF exposure, but there was no increased cancer risk or thermal injuries [65]. Transcranial Electromagnetic Treatment Stops Alzheimer’s Disease Cognitive Decline over a $2\frac {1}{2}$-Year Period: A Pilot Study. Medicines, 9(8), 42). Additionally, 64 MHz has been used for decades in routine RF MRIs without adverse long-term side effects other than acute thermal effects. For this reason, in 2003, the FDA declared “nonsignificant risk status” for MRI clinical systems generating static fields up to 8T or 340 MHz [109].

## XI. Conclusion

This article introduces a new design of a portable Birdcage based meander line antenna for Azheimer’s disease treatments. The design achieves a specific absorption rate (SAR) ranging from 0.4 to 0.9 W/kg within the simulated human brain, focusing on near-field distribution. The HFSS simulations indicate a scattering parameter S11 at 64 MHz while maintaining the antenna size within the portable range. The NEVA head simulation model is employed to compute SAR distribution for the eight-port designs. The SAR values demonstrate greater SAR homogeneity using the proposed design with a 45° phase shift, showcasing potential applications to lower beta amyloid peptide in Alzheimer’s disease treatments. Since the thermal response is considered an important element towards the system stability and patient safety, the numerical NEVA head model was simulated via HFSS with the seven tissue layers of the human head, the simulation ran for an hour to track the temperature change generated by the RF power deposition with a SAR of 0.6 W/kg. The temperature change in millikelvin suggests a future safe exposure during patient treatments in medical settings. Also, given that REMFS mechanism of action is the lowering of A $\beta $ protein levels by activation protein degradation systems, REMFS will become a potential treatment for other protein-associated diseases such as Lewy Body Dementia and Frontal Lobe Dementia.

## Appendix

### Specific Absorption Rate (SAR)

*Definition:* Specific Absorption Rate (SAR) is the time derivative of the incremental energy $ dW $ absorbed by or dissipated in an incremental mass $ dm $ contained in a volume $ dV $ of a given density $ \rho $:

\begin{equation*} SAR = \frac {d}{dt} \left ({{\frac {dW}{dm}}}\right ) = \frac {d}{dt} \left ({{\frac {dW}{\rho dV} }}\right ) \tag {4}\end{equation*}

*Units:* SAR is measured in watts per kilogram (W/kg). It quantifies the amount of radio frequency (RF) energy absorbed by the human body. SAR should be considered an *“absorbed dose rate”* and is related to electric fields at a point by:

\begin{equation*} SAR = \frac {\sigma |E|^{2}}{\rho } \quad \text {(W/kg)} \tag {5}\end{equation*}

where:

• $ \sigma $ is the electrical conductivity (Siemens per meter, S/m),
• $ E $ is the RMS (root mean square) electric field strength (Volts per meter, V/m),
• $ \rho $ is the mass density of the object (kg/m^3^).

*Point SAR (Local SAR):* This equation describes SAR at a single point in the tissue, which can vary over small areas. It is useful for understanding localized heating effects. It is measured using fine-resolution numerical simulations or small probes in experiments and is widely used in medical and bioelectromagnetic research.

*Space-Averaged SAR:* The space-averaged SAR for a body or part of the body is obtained by calculating the local SAR at each point in the body and averaging over the whole body or a specific part.

• **Averaged SAR:** Typically averaged over 1g or 10g of tissue (highest local SAR averaged over 1g or 10g).
• **Whole-Body SAR:** The total power absorbed per unit mass in the entire body. Used for exposure assessments like MRI. Limits for whole-body SAR are:
  • 0.4 W/kg (occupational exposure)
  • 0.08 W/kg (public exposure)

### Integral Form of SAR

The integral form accounts for the spatial distribution of the electric field and provides the total SAR over a given volume of tissue. It calculates the total absorbed power per unit mass and is commonly used in practical applications where SAR is averaged over a small volume (1g or 10g) to assess compliance with safety standards.

\begin{equation*} SAR = \frac {\int _{V} \sigma (r) |E(r)|^{2} dV}{\int _{V} \rho (r) dV} \quad \text {(W/kg)} \tag {6}\end{equation*}

where:

• $ \int _{V} $ denotes the integral over the volume $ V $ of the tissue,
• $ V $ is the volume over which SAR is calculated,
• $ dV $ is a differential volume element,
• $ \sigma (r) $ is the electrical conductivity of the tissue at position $ r $ (S/m),
• $ |E(r)|^{2} $ is the square of the magnitude of the electric field strength (typically RMS) at position $ r $ (V/m),
• $ \rho (r) $ is the mass density at position $ r $ (kg/m^3^).
